# Reconfiguring home language practices in AI-mediated environments: a layered communication perspective on language transmission, affect, and multilingual communication

**DOI:** 10.3389/fpsyg.2026.1859447

**Published:** 2026-05-14

**Authors:** Jun-Yi Chen, Ting-Ting Liu, Tun-Yuan Tien

**Affiliations:** 1 Fuzhou University of International Studies and Trade, Fuzhou, China; 2 Research Center for the Inheritance and Innovation of Handicraft Culture of Humanities and Social Science Base of Fujian Province Universities, Fuzhou, China; 3 Chengdu University of Technology, Chengdu, China; 4 Xiamen University, Xiamen, China

**Keywords:** affect, artificial intelligence-mediated communication (AI-MC), digital communication, home language maintenance, identity, language transmission, masspersonal communication, multilingualism

## Abstract

Home language practices are increasingly reconfigured within AI-mediated environments, where communication may move across interpersonal, mass, and masspersonal conditions rather than remaining within a single communicative form. Under these conditions, conventional distinctions between interpersonal and mass communication remain important, but they do not fully capture how language is produced, circulated, and experienced across private interaction, platform visibility, and algorithmically mediated environments. Drawing on O’Sullivan and Carr’s definition of masspersonal communication as communication that combines high personalization with high message-level accessibility, this article develops a layered communication perspective for understanding how language transmission shifts across interpersonal, mass, and masspersonal configurations. Drawing on oral tradition as a foundational mode of transmission, together with family language practices and digitally mediated interaction, the Tri-Layer Communication Model of AI-Mediated Language Reconfiguration conceptualizes language as moving across interpersonal, mass, and masspersonal contexts. Within this process, AI functions not merely as a tool but as a re-mediating structure that shapes linguistic form, affective expression, and patterns of visibility. Three interrelated dimensions of transformation become visible: the redistribution of affect, the increasing regularization of linguistic forms, and the algorithmic structuring of communication. These transformations reshape how language is perceived, experienced, and sustained, contributing to the emergence of hybrid multilingual practices in which human interaction and technological mediation are deeply intertwined. Home language maintenance in contemporary contexts can therefore be understood as a dynamic process shaped by the interaction of affect, language transmission, and multilingual communication within AI-mediated environments.

## 1 Introduction

### 1.1 Digital transformation and multilingual language practices

In recent years, multilingual language practices have been reshaped by processes of digitalization and platformization. Digital environments are no longer peripheral spaces for language use; instead, they have become central sites where multilingual interaction takes place. Language practices increasingly extend beyond face-to-face communication and unfold across hybrid online–offline contexts. Empirical research on social media shows that users regularly engage in translanguaging across platforms such as Facebook and WhatsApp, blurring conventional language boundaries and drawing flexibly on multiple linguistic resources (Ahmad et al., 2023). Related work on digital storytelling and platform-based learning further demonstrates how technology-mediated environments can integrate diverse linguistic repertoires into participatory practices, thereby supporting multilingual development in new ways (Ferguson-Sams et al., 2024; Garcia, 2026).

Alongside these developments, AI-mediated communication has introduced additional layers of transformation. AI-powered tools, including translation systems and adaptive learning platforms, are increasingly embedded in everyday communicative practices. Studies on AI-assisted translation and language learning suggest that such systems enhance accessibility and provide individualized linguistic support, shaping how multilingual users engage with language across contexts (John and Möller, 2024; Slamet et al., 2025). At the same time, research indicates that AI-mediated communication influences not only what is expressed but also how it is expressed, affecting patterns of linguistic complexity and sentiment (Sussman and Carter, 2025). Rather than functioning solely as tools, these systems participate in the production and structuring of language.

These transformations are not limited to structural aspects of language use. They also extend into affective and identity-related dimensions. Digital and AI-mediated environments influence how multilingual individuals negotiate emotion, identity, and belonging through language. For example, studies of multilingual learners suggest that AI-supported environments can enhance motivation and engagement while reducing communicative anxiety (Ekizer, 2025; Mohebbi, 2025). At the same time, research on multilingual families and migrant communities shows that digital communication practices may both support and challenge the maintenance of heritage languages, reflecting ongoing tensions between linguistic flexibility and preservation (Le, 2025). Multilingualism in the digital age, therefore, is not simply a matter of linguistic competence, but is closely tied to affective experience and social positioning.

### 1.2 The limits of interpersonal and mass communication frameworks

Despite the growing body of research outlined above, studies of language practices continue to be organized around two relatively separate perspectives: interpersonal communication and mass-mediated communication. Within interpersonal contexts, research has focused extensively on family language practices, particularly through the lens of family language policy, heritage language maintenance, and multilingual identity construction. These studies emphasize the roles of parental ideology, child agency, and emotional interaction in shaping language use, highlighting language as both a communicative and affective resource within the family (Gu et al., 2025; Mirvahedi, 2024; Curdt-Christiansen and Iwaniec, 2023). Although recent work acknowledges the presence of digital technologies in family settings, it largely retains an interactional and context-dependent understanding of communication (Li et al., 2026).

In contrast, research on mass-mediated communication examines how language operates within large-scale, platform-based environments. Studies of digital media document the emergence of new forms of multilingual expression, including lexical innovation, identity performance, and cross-cultural interaction (Barrot, 2022; Sun et al., 2021; Hnatyuk et al., 2026). Systematic reviews further indicate that language use in these contexts is shaped by technological affordances and communicative strategies oriented toward visibility and audience reach, rather than immediate interaction (Carilla Pérez and Ruiz Cecilia, 2025; Rapezzi, 2026).

However, an increasing number of communicative practices do not fit neatly within either of these frameworks. AI-mediated systems—such as chatbots, translation tools, and personalized content delivery—are now deeply embedded in everyday communication (Shao et al., 2025; Sussman and Carter, 2025). These forms of interaction are experienced as responsive and individualized, yet are simultaneously enabled by large-scale technological infrastructures. As a result, they blur distinctions between private and public communication, as well as between human and technologically mediated interaction.

What remains underdeveloped in existing research is a systematic way of conceptualizing these hybrid forms. In many cases, they are implicitly categorized as either interpersonal or mass communication, depending on the analytical perspective adopted. This tendency makes it difficult to account for communicative processes that combine personalization, interactivity, message-level accessibility, and technologically mediated infrastructure. The result is a conceptual gap in understanding how language operates in environments where communication is both individualized and infrastructurally mediated.

### 1.3 A layered communication perspective and research aim

To address this gap, this study draws on the concept of masspersonal communication, which O’Sullivan and Carr define in terms of the combination of high personalization and high message-level accessibility (O’Sullivan and Carr, 2018). In this framework, accessibility is not a secondary or infrastructural feature, but a defining condition of masspersonal communication. A communication practice may be highly personalized, but if the specific message remains accessible only to the immediate participants, it should not be classified as masspersonal communication. This distinction is especially important for AI-mediated communication. AI systems may generate personalized responses at scale, but system-level scalability alone does not make an interaction masspersonal. A private AI conversation, a private AI translation, or a smart reply used only within a dyadic exchange remains primarily interpersonal-dominant unless the resulting message becomes accessible beyond the immediate participants. Masspersonal communication becomes analytically relevant only when personalization is combined with message-level accessibility, such as persistence, shareability, public or semi-public visibility, or algorithmically organized availability to broader audiences. The present article therefore does not treat AI-mediated communication as automatically masspersonal. Instead, it develops a layered communication perspective in which interpersonal, mass, and masspersonal configurations may become dominant under different communicative conditions.

Subsequent research has shown that the boundaries between interpersonal and mass communication have become increasingly fluid in digitally mediated environments. The convergence of communication technologies has reshaped key dimensions of communication, including audience scale, directionality, and feedback processes (Flanagin, 2017; Walther, 2017; Walther and Valkenburg, 2017). Within this context, the concept of masspersonal communication offers a useful way of capturing forms of interaction that cannot be adequately explained by traditional binary models.

This perspective becomes particularly relevant in the context of AI-mediated communication. Existing research suggests that AI systems may be perceived as socially meaningful actors when they display responsiveness, human-like cues, or forms of machine agency that invite users to orient to them as communicative partners rather than as neutral tools (Westerman et al., 2020; Ngo, 2026; Poinsot et al., 2022). This perceived sociality does not depend on attributing intrinsic sentience or moral status to AI. Rather, it can be understood as emerging from cognitive mechanisms that structure human social perception, including agency detection, theory of mind, and sensitivity to relational continuity, all of which can be activated in interaction with increasingly fluent AI systems (Ryu, 2026).

This perspective helps clarify why AI may be conceptualized as a re-mediating structure or quasi-other within AI-mediated communicative environments. AI does not participate in communication in the same way as human interlocutors, yet it may still become interactionally salient enough to shape how language, affect, and relational orientation are organized. In this sense, AI is not merely a channel through which communication passes, but a socially consequential presence embedded in communicative processes (Negura, 2025; Boyd and Markowitz, 2026; Ryu, 2026).

These characteristics also help explain why AI-mediated systems can reproduce features commonly associated with interpersonal communication, such as responsiveness, emotional expression, and perceived reciprocity, while still operating within large-scale technological infrastructures. For this reason, AI-mediated communication should be understood not only in terms of message transmission, but also in terms of how machine agency and perceived social presence reshape the conditions under which communication is interpreted and experienced (Mieczkowski et al., 2021; Brandtzaeg et al., 2023; Saile and Hüttl-Maack, 2026).

Against this background, this study situates itself at the intersection of language transmission, communication theory, and AI-mediated environments. It takes oral tradition as a starting point for understanding language transmission as an embodied and relational process, conceptualizes family language as a dynamic communicative practice, and considers AI as a structural condition that reshapes how communication unfolds.

The aim of this study is to develop a conceptual framework for understanding how language transmission is reconfigured across communicative layers in AI-mediated environments. In particular, it examines how oral transmission is transformed within a layered communication framework, and how AI-mediated processes reshape both the circulation of language and its associated affective dimensions.

## 2 From oral tradition to layered communication

Language transmission is often described through distinct domains, yet in practice these domains overlap and evolve over time. What appears as oral tradition, family language, and digitally mediated communication can be understood as different phases within a broader process rather than isolated phenomena.

### 2.1 Oral tradition as a situated mode of transmission

Oral tradition offers a way to observe language in use at its most immediate level. Language is not simply transferred through exposure; it takes shape through repeated interaction, where speakers respond to one another and gradually establish shared patterns. Studies of family interaction and spoken communication show that linguistic forms are constructed within ongoing social relationships rather than learned in isolation (Gordon, 2009; Valentinsson, 2018).

Such processes depend on participation. Everyday interaction, including mealtime conversations and storytelling, does more than convey information. It creates conditions under which language becomes recognizable and repeatable. Turn-taking, repetition, and collaborative meaning-making contribute to the emergence of shared linguistic patterns, sometimes described as “familylects” (Valentinsson, 2018; Gordon, 2009). These patterns stabilize through use rather than formal instruction.

Emotional engagement and contextual variation shape how these patterns are sustained and transformed. Storytelling often carries both narrative structure and affective weight, supporting language development alongside relational bonding (Kellas, 2005; Zhang et al., 2026). Intergenerational interaction extends this process, particularly where grandparents contribute to heritage language maintenance by linking language to familiarity and belonging (Lubis, 2024; Fatima and Nadeem, 2025). At the same time, narrative forms vary across cultural settings, influencing how stories are organized and interpreted (Makazhu et al., 2026; Tulviste, 2019). Oral practices such as proverbs, songs, and storytelling carry cultural knowledge and collective memory alongside linguistic forms (Almiron et al., 2024; Person, 2015). Language transmission therefore remains inseparable from the environments in which it is embedded.

### 2.2 Family language as an ongoing communicative practice

If oral transmission highlights interaction as a starting point, family language shows how this interaction is sustained and reshaped over time. Within families, language does not operate as a fixed system. It is continuously negotiated through everyday communication, often in ways that remain implicit.

Research on multilingual families indicates that language use adapts to shifting circumstances. Practices such as translanguaging, code-switching, and informal instruction are embedded in daily routines and emerge in response to changing linguistic environments (Gu et al., 2026; Kheirkhah and Cekaite, 2015; Fatima and Nadeem, 2025). These practices reflect ongoing adjustment between participants rather than predetermined rules.

Language policy within families rarely functions as a stable framework. Even when parents express clear preferences, outcomes depend on how these preferences are interpreted, negotiated, or resisted. Children influence these processes through participation, acceptance, or refusal, shaping language use alongside adults (Romanowski, 2021; Mazzaferro, 2018; Kheirkhah and Cekaite, 2015; Sitareniou and Mousoulidou, 2026). Reviews of family language research suggest that what appears as policy often emerges through ongoing adjustment rather than prior design (Chen and Cao, 2025).

Emotional dynamics remain closely involved. Feelings such as attachment, anxiety, or pride can influence language choice and long-term maintenance (Wang, 2023; Hatoss, 2025). Heritage language use is often tied to identity formation and intergenerational connection, reinforcing cultural belonging within family contexts (Alshihry, 2024; Lubis, 2024). Family language is therefore better understood as a practice that unfolds through interaction, shaped by negotiation and affect rather than governed by fixed rules.

### 2.3 Communication as a layered system

When these dynamics are considered alongside developments in digital media, it becomes increasingly difficult to describe communication within a single framework. The distinction between interpersonal and mass communication still captures important differences, but it no longer accounts for the full range of communicative practices that have emerged.

Interpersonal communication remains grounded in reciprocal interaction, where meaning develops through ongoing exchange (Podschuweit, 2017). At the same time, mass communication continues to structure language at scale, shaping how information circulates across large audiences (Flanagin, 2017). What has changed is the extent to which these modes intersect.

The concept of masspersonal communication captures this overlap by pointing to forms of interaction that are both highly personalized and accessible beyond the immediate participants (O’Sullivan and Carr, 2018; Walther, 2017). This overlap should not be understood as a stable midpoint between interpersonal and mass communication. Instead, the relative influence of each layer varies according to communicative conditions. When responsiveness, audience specificity, and perceived intimacy are foregrounded within private interaction, communication may function in a more interpersonal-like way. When visibility, dissemination, and broad circulation become more central, mass communication exerts stronger influence. Masspersonal communication becomes analytically important only when personalization is combined with message-level accessibility, allowing communication to be experienced as individually relevant while also remaining available beyond the dyadic exchange (Flanagin, 2017; O’Sullivan and Carr, 2018). In AI-mediated environments, this distinction is especially important because responsive or individualized interaction does not by itself constitute masspersonal communication. Chatbots, recommendation systems, and similar technologies may generate, filter, or adapt communication through large-scale infrastructures, but the masspersonal layer becomes relevant only when specific communicative outputs are also persistent, shareable, publicly or semi-publicly visible, or algorithmically available beyond the immediate interaction.

### 2.4 AI as a re-mediating structure

These shifts cannot be fully understood if technology is treated as a neutral medium. AI systems intervene in communication in ways that reshape how language is produced, interpreted, and circulated (Sundar, 2020; Hancock et al., 2020).

Changes in linguistic form are one indication of this intervention. AI-generated communication often shows patterns that differ from human-produced language, including shifts in sentiment and simplification of structure (Sussman and Carter, 2025). Tools such as translation systems and chatbots make communication more efficient, but they also introduce regularities that were not previously present (Yang et al., 2023; Fu et al., 2024). At the same time, these systems respond to emotional cues and produce expressions that resemble empathy, influencing how affect is expressed and perceived in interaction (Rao et al., 2026). Such responses may be interpreted differently from human communication, particularly when their artificial origin is recognized (Kober et al., 2026), and they complicate questions of trust and attribution by distributing emotional meaning across human and technological actors (Hohenstein and Jung, 2020).

Visibility is shaped in comparable ways. Algorithmic systems influence which forms of language are encountered, affecting patterns of exposure and engagement (Sussman et al., 2026; Shafik, 2026). These processes extend beyond individual interaction and shape broader communicative environments, where some forms of language are amplified while others remain less visible (Nisha et al., 2026).

What emerges from these shifts is a situation in which AI does not simply support communication but reorganizes the conditions under which it takes place. Language continues to move across interpersonal, mass, and masspersonal contexts, yet the pathways it follows are increasingly structured by technological systems. This provides the basis for the conceptual model developed in the next section.

At the same time, the role of AI in language transmission should not be assumed to be uniform across all participants or stages of development. This is especially important in early language acquisition contexts, where children’s speech is often incomplete, non-standard, and context-dependent, and where current AI and ASR systems continue to face substantial limitations due to physiological, cognitive, and extrinsic factors (Singh V. P. et al., 2025; McGonigle et al., 2024; Southwell et al., 2022). These constraints suggest that AI may not always function effectively as a fully responsive conversational partner for young children. In such settings, it may be more analytically appropriate to distinguish between AI as a direct conversational partner and AI as an indirect environmental or infrastructural mediator that organizes learning activities, scaffolds interaction, or shapes the communicative conditions under which language practice occurs (Quan et al., 2026; Xiao et al., 2025; Ji et al., 2023; Gazit, 2025).

## 3 A conceptual model of AI-mediated language reconfiguration

### 3.1 Model overview

The preceding discussion points to a set of communicative processes that are difficult to capture within existing models. Language does not remain confined to a single domain, nor does it move unchanged between contexts. Instead, it shifts as it circulates, taking on different forms depending on the conditions under which it is produced and interpreted.

**Figure 1:**
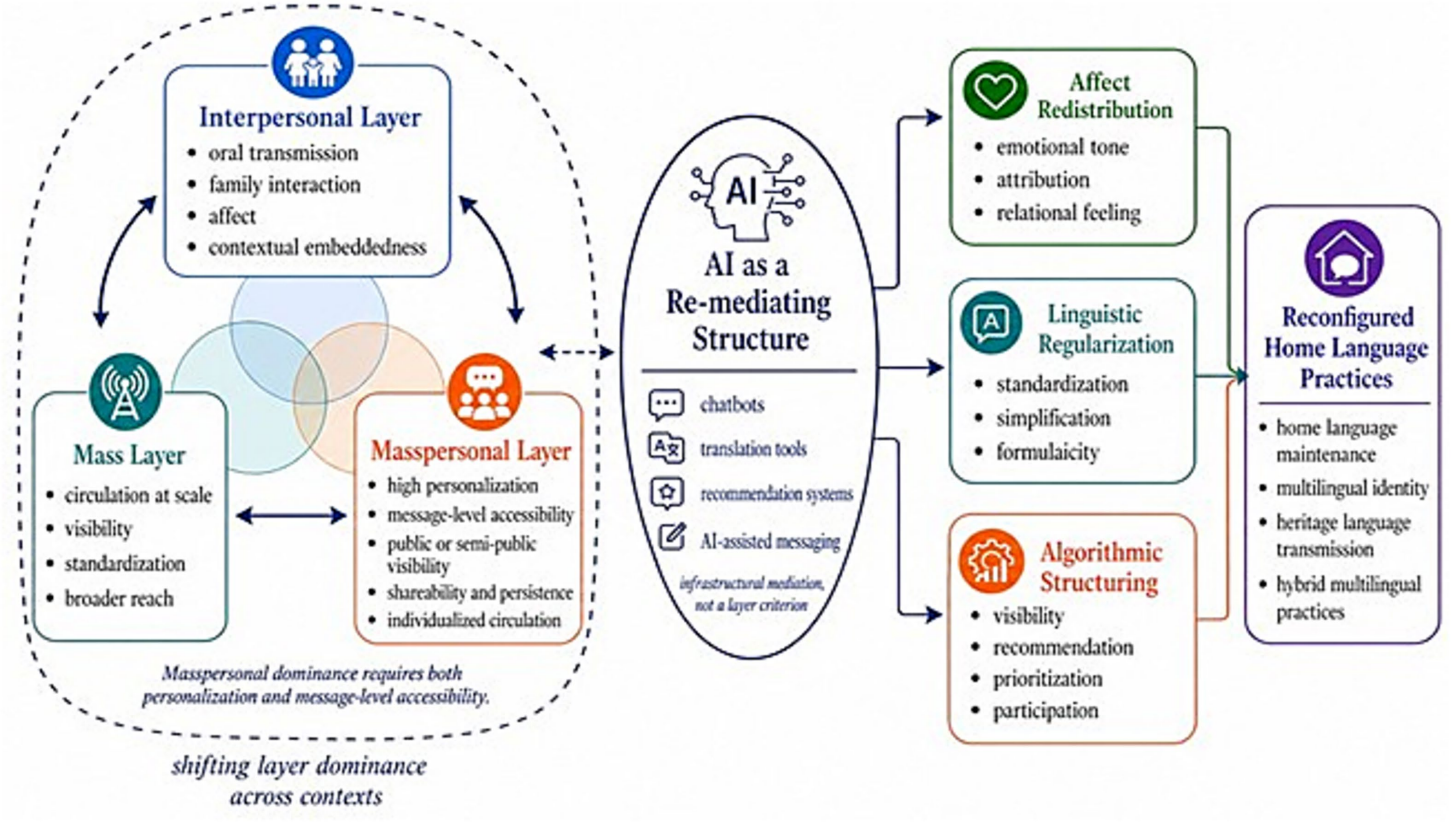
The tri-layer communication model of AI-mediated language reconfiguration. The model illustrates how home language practices may be reconfigured through the shifting dominance of interpersonal, mass, and masspersonal communication layers in AI-mediated environments. The dashed boundary on the left indicates that these layers are not fixed stages, but contextually activated and unevenly dominant communicative conditions. In line with O’Sullivan and Carr’s framework, the masspersonal layer is defined by the combination of high personalization and high message-level accessibility. AI-mediated interaction is therefore not treated as automatically masspersonal. Private chatbot use, private AI translation, and smart replies remain interpersonal-dominant when their outputs are accessible only to the immediate participants. The masspersonal layer becomes dominant only when message-level personalization is combined with persistence, shareability, public or semi-public visibility, or algorithmic accessibility beyond the dyadic exchange. AI functions as a re-mediating structure that may shape communication through affect redistribution, linguistic regularization, and algorithmic structuring, with implications for home language maintenance, multilingual identity, heritage language transmission, and hybrid multilingual practices.

The Tri-Layer Communication Model of AI-Mediated Language Reconfiguration is proposed to account for this movement. It approaches language transmission as a process unfolding across three interconnected layers: interpersonal, mass, and masspersonal communication. These layers are not discrete stages but overlapping conditions within which language is continuously shaped. Figure 1 presents the Tri-Layer Communication Model of AI-Mediated Language Reconfiguration. The figure visualizes the model as a layered and non-linear process rather than a sequential movement from one communicative form to another. The interpersonal, mass, and masspersonal layers are shown as mutually interacting conditions whose relative dominance shifts across contexts. The dashed boundary indicates this contextual shifting, while the solid arrows among the layers emphasize their reciprocal influence. The dashed bidirectional connector between the layer cluster and AI further indicates that AI-mediated structures do not simply act upon communication from outside; rather, they are shaped by existing communicative practices while also reorganizing the conditions under which language is produced, circulated, and interpreted. This visual representation clarifies the model’s internal logic by showing how communicative layers, AI-mediated structures, transformation dimensions, and language-practice outcomes are analytically connected.

Interpersonal communication provides the setting in which language emerges through interaction, shaped by affect and contextual embeddedness. Mass communication extends this process by enabling language to circulate at scale, introducing standardization and broader reach. Masspersonal communication introduces a different configuration in which communication is both highly personalized and highly accessible at the message level (O’Sullivan and Carr, 2018; Walther, 2017). In the present model, system-level scalability is treated as a feature of AI-mediated infrastructure rather than as a defining criterion of masspersonal communication.

As language enters AI-mediated environments, different communicative configurations may become active, including interpersonal, mass, and masspersonal conditions. AI-mediated systems do not simply transmit language but participate in shaping it, influencing how expressions are generated, distributed, and interpreted. What follows is not a linear transition from one form to another, but an ongoing reconfiguration in which language is continuously adjusted through interaction with technological systems. As illustrated in Figure 1, the three communicative layers do not exert equal influence at all times. Their relative force depends on the conditions under which communication unfolds, including the degree of personalization, the scope of accessibility, the scale of visibility, and the communicative affordances of the platform or system involved. The model therefore treats interpersonal, mass, and masspersonal communication not simply as co-present layers, but as unevenly activated dimensions whose influence shifts across contexts (Flanagin, 2017; O’Sullivan and Carr, 2018; Walther, 2017). Crucially, the masspersonal layer is not activated simply because AI is involved; it becomes dominant only when personalized communication also has message-level accessibility beyond the immediate dyadic exchange.

### 3.2 Key dimensions of transformation

To make the model more analytically precise, the three dimensions identified here can be understood as conceptually distinct but related forms of transformation. Affect redistribution refers to shifts in how emotional expression, perception, attribution, and relational feeling are distributed across human interlocutors and AI-mediated systems. Linguistic regularization refers to the reduction of variability in linguistic output through processes such as standardization, simplification, formulaicity, or optimization for clarity. Algorithmic structuring refers to the ways algorithmic systems shape exposure, visibility, recommendation, prioritization, and participation in communication environments (Hohenstein and Jung, 2020; Mieczkowski et al., 2021; Ferdinand et al., 2019; Casapa and Puschmann, 2025; Jones, 2021).

Affect redistribution becomes visible when communication is examined across different layers. AI-mediated communication does not simply add emotional cues to interaction; it redistributes how affect is expressed, perceived, and attributed across human participants and technological systems. AI-generated or AI-assisted responses may shape emotional tone, reinforce particular affective patterns, or alter how relational intent is interpreted in interaction. In this sense, affect is not removed from communication, but reorganized through mediation (Hohenstein and Jung, 2020; Jakesch et al., 2019; Mieczkowski et al., 2021).

At the same time, such expressions are not necessarily experienced in the same way as those produced in interpersonal contexts. Existing work suggests that AI involvement can reshape trust, authenticity judgments, and emotional accountability, so that relational feeling may remain present while becoming less clearly anchored in a single human source (Hohenstein and Jung, 2020; Jakesch et al., 2019).

Linguistic regularization points to a related shift in communicative form. In the present framework, linguistic regularization refers to the reduction of variability in language through standardization, simplification, formulaic patterning, or optimization for clarity. AI-mediated communication often favors outputs that are easier to process, more predictable in structure, and more consistent across contexts. Such regularization can support accessibility and communicative efficiency, but it may also narrow stylistic variation and reduce linguistic flexibility over time (Ferdinand et al., 2019; Krishna et al., 2025; Getino-Diez and García-Madariaga, 2026; Mitra et al., 2025).

This creates a tension within AI-mediated communicative environments: communication can be tailored to individuals, while the structures that enable such personalization may still rely on standardized linguistic patterns.

Algorithmic structuring becomes visible at the level of communicative organization. In this framework, algorithmic structuring refers to the ways algorithmic systems shape exposure, visibility, recommendation, prioritization, and participation in communication environments. Language is not simply exchanged or broadcast; it is increasingly filtered, ranked, and circulated through systems that influence what becomes more visible, what remains peripheral, and how users are positioned within communicative processes. These dynamics affect not only access to content, but also the conditions under which language is encountered, repeated, and taken up in practice (Casapa and Puschmann, 2025; Jones, 2021; Smets et al., 2021; Yeung, 2018).

Although the present study remains conceptual, these three dimensions also indicate possible directions for future empirical work. Affect redistribution may be examined through changes in emotional tone, attribution, trust, or perceived relational authenticity; linguistic regularization through reduced lexical or structural variability and increased formulaicity; and algorithmic structuring through patterns of visibility, recommendation, prioritization, and participation across communication environments (Hohenstein and Jung, 2020; Mieczkowski et al., 2021; Ferdinand et al., 2019; Casapa and Puschmann, 2025; Jones, 2021).

### 3.3 Layer dominance in AI-mediated communication

The model can be clarified further by specifying not only how interpersonal, mass, and masspersonal layers coexist, but also the conditions under which one layer becomes analytically dominant. In this framework, layer dominance does not mean that the other layers disappear. Rather, it refers to the communicative logic that most strongly organizes a given interaction at a particular moment. The relative dominance of a layer depends on several communicative conditions, including reciprocal responsiveness, audience specificity, message-level accessibility, platform visibility, and the extent to which algorithmic infrastructures shape persistence, recommendation, or distribution (O’Sullivan and Carr, 2018; Walther, 2017). For the purposes of the present model, system-level scalability is treated as an infrastructural feature of AI-mediated environments rather than as a defining condition of masspersonal communication. A further distinction is also necessary between message-level personalization and exposure-level personalization. Message-level personalization refers to cases in which the communicative content itself is tailored to a specific user, relationship, prompt, or interactional context. Exposure-level personalization refers to cases in which the same message is distributed or recommended differently across users, while the message content itself remains unchanged.

This clarification is particularly important for AI-mediated communication. AI-mediated communication should not be treated as automatically masspersonal. A private conversation between a child and an AI system, for example, may remain primarily interpersonal-dominant if the interaction is dyadic, private, reciprocal, and context-specific. In such a case, AI may function as a responsive conversational partner or quasi-interlocutor, but the communicative process is organized mainly by immediacy, turn-taking, perceived responsiveness, and situated interaction rather than by public accessibility. This point is consistent with the broader understanding of AI-mediated communication as communication that may augment, modify, or generate messages within particular interactional contexts, rather than as a single fixed communicative form (Hancock et al., 2020; Mieczkowski et al., 2021; Meng et al., 2025).

For this reason, it is necessary to distinguish carefully between message-level accessibility and system-level scalability. Message-level accessibility refers to whether a specific communicative output is available beyond the immediate participants. System-level scalability refers to whether the communicative infrastructure can be deployed repeatedly across users, contexts, and situations. However, system-level scalability is not a defining criterion of masspersonal communication. In line with O’Sullivan and Carr’s framework, masspersonal communication requires both high personalization and high message-level accessibility. A private AI conversation may be personalized and technologically scalable, but if the communicative output remains accessible only to the immediate user, it should be classified as interpersonal-dominant AI-mediated communication rather than masspersonal communication. System-level scalability may shape the conditions of interaction, but it does not by itself transform a private exchange into masspersonal communication.

The masspersonal layer becomes dominant only when individualized communicative experience is combined with message-level accessibility beyond the immediate participants. This differs from simply saying that personalization and accessibility coexist. The analytical issue is whether personalization remains confined to a private exchange or whether it becomes linked to broader systems of persistence, repeatability, recommendation, ranking, or circulation. When communication is experienced as individually tailored while also becoming accessible beyond the immediate participants through persistence, shareability, public or semi-public visibility, or algorithmic availability, the masspersonal layer becomes more analytically salient.

A more fine-grained classification of AI-mediated communication can therefore be proposed. First, direct conversational AI interaction is usually interpersonal-dominant when it remains private, reciprocal, and context-specific. Such interaction may be shaped by scalable infrastructure, but it should not be described as masspersonal unless the message itself becomes accessible beyond the immediate participants. Second, AI-assisted interpersonal communication, such as suggested replies, AI-guided support messages, tone-adjustment systems, or translation support, should not be treated as masspersonal by default. These tools may shape interpersonal communication by modifying, augmenting, or generating message content for communicative and relational purposes, including suggested text responses and AI-assisted support messages (Mieczkowski et al., 2021; Meng et al., 2025). However, when these tools operate within private dyadic exchanges, they remain primarily interpersonal-dominant because the resulting message is accessible only to the immediate participants. In line with O’Sullivan and Carr’s definition of masspersonal communication, they become relevant to masspersonal communication only when the AI-assisted message is not only personalized but also persistent, shareable, publicly or semi-publicly visible, or algorithmically available beyond the original dyadic exchange (O’Sullivan and Carr, 2018). Third, algorithmically mediated public or semi-public content should be differentiated according to whether personalization occurs at the level of the message itself or only at the level of exposure. AI-generated public posts, such as a heritage-language story, caption, or short text produced in response to a child’s or parent’s prompt and then shared beyond the immediate interaction, represent a stronger example of masspersonal communication because the message itself is personalized while also becoming accessible to others. By contrast, recommended videos or platform-curated multilingual materials may remain closer to mass communication with personalized distribution when the message content itself is identical across users and only the exposure pathway is individualized. In such cases, the masspersonal layer should not be assumed to dominate unless the message itself is personalized and also accessible beyond the immediate participants.

This classification also clarifies why AI-mediated environments are significant for home language practices. In family settings, AI may operate as a direct conversational partner, an assistive tool for translation or writing, a platform mechanism that recommends language content, or an infrastructural mediator that shapes what linguistic forms become visible and repeatable. These roles do not map onto a single communicative layer. Instead, they activate different layer configurations. A child privately practicing a heritage language with a chatbot may be situated in an interpersonal-dominant configuration because the exchange is dyadic, responsive, and not accessible beyond the immediate interaction. A parent using AI translation to communicate privately with a child also remains interpersonal-dominant, even though the exchange is shaped by AI-mediated linguistic support. By contrast, a family encountering algorithmically recommended heritage-language content online may be situated in a mass-dominant configuration with personalized exposure if the same content is simply recommended differently to different users. Such a case should be distinguished from masspersonal communication, where the message itself is personalized and also accessible beyond the immediate participants. For example, if a parent or child uses AI to generate a heritage-language story, caption, or public post based on a specific family context and then shares it through a platform, the case more clearly activates the masspersonal layer because message-level personalization and message-level accessibility are both present.

The theoretical value of the tri-layer model therefore lies in its ability to identify shifting communicative dominance rather than to assign AI-mediated communication to one fixed category. AI intensifies the need for a layered approach because it can simulate interpersonal responsiveness, operate through mass-scale infrastructures, and personalize communicative outputs at the same time. The masspersonal layer becomes analytically salient not whenever AI is present, but when individualized communication is also accessible beyond the immediate participants through persistence, shareability, public or semi-public visibility, or algorithmic availability.

To make this layered logic more practically visible, several illustrative scenarios may be considered. A child privately practicing a home or heritage language with a conversational AI system may remain in an interpersonal-dominant configuration because the exchange is dyadic, responsive, and context-specific. At the same time, the interaction is still shaped by a scalable AI infrastructure capable of producing similar individualized exchanges across different users and settings. A parent using AI translation or tone-adjustment tools to communicate privately with a child should be treated as interpersonal-dominant rather than masspersonal, unless the AI-assisted message becomes accessible beyond the immediate participants. Similarly, a family encountering language-learning materials, songs, stories, or multilingual content through platform recommendation systems may represent mass communication with personalized exposure when the same content is recommended differently across users. It becomes masspersonal only when the message itself is personalized, such as when AI generates a family-specific heritage-language story or caption that is subsequently shared or made visible beyond the original dyadic interaction. These examples show that the model is not intended to classify technologies as fixed communicative forms, but to identify how layer dominance shifts across specific home language practices.

## 4 Reconfiguring family language in AI-mediated environments

### 4.1 From embodied Oral practice to mediated expression

Family language has traditionally been sustained through embodied interaction, where communication unfolds in shared physical space. Meaning is not conveyed through words alone but through gaze, gesture, and the broader context of co-presence. These elements support synchrony and contribute to emotional attunement within family interaction (Magni et al., 2025; Osler and Zahavi, 2023).

As communication increasingly moves across digital environments, this form of interaction begins to shift. Family members often alternate between face-to-face and mediated communication, sometimes within the same setting, reflecting an expanded communicative repertoire (Stæhr and Nørreby, 2021). Digital tools allow interaction to continue across distance, yet the absence of embodied cues changes how meaning is conveyed and interpreted (Burholt et al., 2020).

These changes affect not only how communication occurs but also its depth and texture. Studies suggest that reliance on mediated interaction may be associated with more fragmented patterns of engagement, where interaction becomes less continuous and more episodic (Dumbadze and Metreveli, 2025; Kansal et al., 2025). In multilingual and transnational families, this shift can also reshape language use by reinforcing dominant societal languages and altering intergenerational transmission patterns (Kheirkhah and Cekaite, 2018; Chen and Liu, 2025).

At the same time, communication does not simply diminish under these conditions. Family members adjust by developing alternative strategies. In the absence of embodied cues, greater reliance is placed on explicit expression and symbolic resources, which changes how meaning is constructed in interaction (Zulkifli and Roslan, 2026). What emerges is not a replacement of oral practice but a redistribution of communicative resources across different modes.

### 4.2 Simulated affect and algorithmic intimacy

Affective expression within family language is also being reshaped as AI-mediated communication becomes more common. In interpersonal contexts, emotion is grounded in shared experience and relational history. AI systems introduce different mechanisms through which affect can be expressed and interpreted.

Research shows that AI systems can generate responses that align with emotional cues, using forms of artificial empathy that incorporate tone adjustment, linguistic mirroring, and context-sensitive replies (Rao et al., 2026; Xiang et al., 2025). These features can support engagement and create a sense of responsiveness in interaction. In certain contexts, such as conversational agents, users may even experience reduced loneliness or increased emotional comfort (Kim et al., 2025; Liu et al., 2026).

The nature of this engagement, however, remains distinct from interpersonal emotion. Studies consistently indicate that AI-generated responses are often perceived as less authentic, particularly when users are aware of their artificial origin (Hao et al., 2025; Shen et al., 2024; Rubin et al., 2025). Emotional expression may resemble empathy in form, yet it is interpreted through a different frame of reference.

At the same time, these systems are designed in ways that foster a sense of closeness. Personalization, responsiveness, and interactional feedback can lead users to experience a form of intimacy that emerges from system design rather than shared human experience (Al Farisi et al., 2022; Kang, 2026). Processes such as emotional mirroring and linguistic reflection further reinforce this perception, allowing AI responses to be interpreted as empathetic even when they are generated algorithmically (Diaz et al., 2026).

This combination produces a form of intimacy that is both accessible and contingent. Emotional connection becomes possible in new ways, yet remains shaped by technological mediation. Within family contexts, this suggests that affect is increasingly co-produced through interaction that involves both human participants and AI systems, altering how emotional meaning is experienced and negotiated.

### 4.3 Hybridization of language practices

As AI systems become more integrated into everyday communication, family language practices take on increasingly hybrid forms. Interaction is no longer confined to direct exchanges between individuals but unfolds through configurations that involve both human participants and technological intermediaries.

Research on AI-mediated communication shows that these systems can influence interaction in multiple ways. Tools such as AI-assisted messaging can shape conversational tone and affect how participants evaluate one another, including perceptions of trust and relational intent (Mieczkowski et al., 2021). In some situations, AI may absorb communicative tension or redirect interpretation, functioning as an intermediary that reshapes relational dynamics (Hohenstein and Jung, 2020).

These effects extend beyond individual exchanges. Algorithmic systems can introduce consistent patterns into communication, such as tendencies toward positivity or standardization, which influence how users coordinate interaction and respond to one another (Mieczkowski et al., 2021). In collaborative settings, AI-generated suggestions may facilitate alignment and coordination, supporting interaction while also altering its structure (Hall et al., 2025). Within family contexts, this means that communication is increasingly shaped by system-generated cues alongside interpersonal negotiation.

At the same time, language becomes a site where human intention and technological mediation intersect. AI systems embedded in digital platforms can participate in meaning-making processes, influencing how identity is expressed and interpreted through features such as translation, voice synthesis, and adaptive interaction (Yin et al., 2025). In multilingual families, these processes can affect language choice and the ways in which linguistic identity is negotiated across contexts.

This hybridization introduces both possibilities and tensions. While AI-mediated communication can enhance accessibility and support coordination, it may also raise questions about authenticity, emotional connection, relational depth, and the role of algorithmic influence in interaction (Singh J. et al., 2025; Yaacob et al., 2025). Communication can feel simultaneously personalized and mediated, reflecting a balance that is not easily stabilized.

### 4.4 Implications for language maintenance and identity

These transformations have implications for how language is maintained and how identity is formed within family contexts. Language transmission no longer takes place solely within the boundaries of the household. It extends across digital environments that connect family members to broader networks.

Digital communication can support language maintenance by enabling interaction across distance. Younger generations may engage with extended family members and maintain contact with heritage language environments through mediated communication, contributing to intergenerational continuity (Ringblom et al., 2024). These processes, however, remain dependent on participation and do not operate independently of family interaction.

Identity is also shaped within these expanded environments. Digital platforms provide spaces in which individuals draw on multiple linguistic and cultural resources, allowing for the expression of hybrid identities (Palacios-Hidalgo and Huertas-Abril, 2025). Language use in these contexts contributes to how individuals position themselves socially, both within families and in relation to broader communities (Alasal, 2025).

Changes in linguistic practice further illustrate this shift. Digital environments support flexible language use, including code-switching and translanguaging, enabling speakers to adapt their linguistic choices across contexts (Yousif, 2025; Ng and Lee, 2019). At the same time, platform-specific conventions such as abbreviated forms, symbolic expression, and stylistic variation influence how language is produced and interpreted (Dekalo et al., 2025; Kurbanbaev and Baxtiyorov, 2025).

Language maintenance and identity formation therefore emerge through processes that extend beyond direct interaction. They are shaped by the interplay between interpersonal communication and digitally mediated environments, where access, visibility, and technological structures influence how language is used and sustained.

## 5 Discussion

### 5.1 Theoretical contributions

This study brings together several strands of research that are often treated separately and shows how they can be read within a single analytical perspective. Rather than introducing entirely new concepts, its contribution lies in reorganizing existing ones in a way that makes ongoing transformations in language practices more visible.

The starting point is a reconsideration of oral tradition. Instead of treating it as a cultural residue, the analysis approaches it as a mode of transmission grounded in interaction, affect, and shared context. This makes it possible to trace how language begins within situated communication before moving into increasingly mediated environments.

From this position, family language can be understood in a different way. Rather than a bounded system or a set of stable practices, it appears as something that is continually negotiated. What happens within families reflects not only interpersonal relations but also broader communicative conditions. This shift makes family language a useful site for observing how larger transformations take shape at the level of everyday interaction.

The discussion then extends to communication itself. The distinction between interpersonal and mass communication remains relevant, but it is no longer sufficient. By introducing a layered perspective, the study shows how communication moves across different conditions rather than remaining fixed within one domain. Masspersonal communication becomes relevant in this regard, not as a general label for AI-mediated communication, but as a specific configuration in which personalization is combined with message-level accessibility.

Within this layered framework, AI is not treated as an external factor but as something that reshapes the conditions of communication. It affects how language is generated, how affect is expressed, and how visibility is organized. The notion of re-mediation clarifies this role by emphasizing how technological systems intervene in the movement of language across communicative layers.

These elements come together in the Tri-Layer Communication Model of AI-Mediated Language Reconfiguration. The model does not describe communication as a stable structure, but as a set of processes through which language is continuously reconfigured. What emerges is a view of language that is distributed across layers and shaped through ongoing interaction with technological systems.

### 5.2 Implications for multilingualism in the digital age

Multilingualism in the digital age can no longer be understood simply as the coexistence of multiple languages. It takes shape within communicative environments where interaction, mediation, and technological systems are closely intertwined.

One implication concerns affect. Emotional expression is no longer confined to interpersonal interaction but is increasingly shaped by mediated environments in which meaning is produced and interpreted across human and technological systems. Multilingual speakers move between linguistic resources while also engaging with AI-mediated forms of interaction, which alters how affect is experienced and expressed.

Another implication relates to digital inequality and linguistic homogenization. Digital environments expand possibilities for multilingual expression, yet these possibilities are unevenly distributed. Access to technologies, visibility within platforms, and the distribution of communicative resources influence how language can be used, recognized, and sustained. This inequality is not only a matter of access to devices or platforms, but also a matter of linguistic representation within AI-mediated systems. Existing research suggests that language technologies and AI systems may privilege dominant, standardized, or high-resource languages, while languages and varieties with fewer digital resources may remain less visible, less accurately processed, or less effectively supported (Albeihi and Rice, 2025; Helm et al., 2024; Yin, 2025). This issue is closely connected to the risk of linguistic homogenization. When AI-mediated tools rely on standardized language models, translation norms, or dominant-language datasets, they may encourage users toward more regularized and widely represented linguistic forms. Such processes may support communicative efficiency, but they can also narrow the space for dialectal variation, heritage language practices, and mixed multilingual expression. Research on language technology bias and local languages further suggests that these systems may reproduce forms of epistemic or linguistic inequality when they fail to adequately recognize diverse linguistic communities and locally situated forms of meaning-making (Eriksson Krutrök and Poromaa Isling, 2025; Helm et al., 2024; Yin, 2025). For home language maintenance, this means that AI-mediated support cannot be evaluated only by whether it increases access to language resources. It must also be examined in terms of which linguistic forms are made visible, which are regularized, and which are rendered peripheral within AI-mediated environments.

More broadly, multilingual practices appear as dynamic rather than stable. Language is not maintained within a single domain but emerges through movement across different communicative layers. In this process, AI-mediated systems function as conditions that reorganize how language is produced and experienced. Multilingualism becomes a hybrid phenomenon shaped by the interaction between speakers, communicative structures, and technological mediation.

### 5.3 Future directions

The framework developed here opens several directions for further work. One concerns the role of linguistic diversity under conditions of unequal visibility. Different languages occupy different positions within digital environments, and examining how these positions affect movement across communicative layers would extend the current analysis.

Another direction involves the operation of algorithmic systems. While this study conceptualizes AI as a re-mediating structure, further research is needed to examine how such systems prioritize, filter, and reshape language in practice. This includes questions of representation, amplification, and the distribution of communicative attention.

How this framework plays out in specific contexts remains an open question. This is especially true in early language acquisition settings, where the communicative role of AI may be constrained by the current limitations of child speech recognition and spoken language understanding. Children’s speech is often incomplete, variable, and context-dependent, which makes it difficult for AI systems to function consistently as fully responsive interlocutors. These limitations are especially important for home and heritage language transmission. Children’s speech may differ from adult linguistic norms because of developmental variation, unstable pronunciation, mixed-language use, and context-dependent meaning-making. When AI systems fail to recognize or appropriately respond to such speech, the problem is not only technical. Misrecognition may interrupt interaction, reduce children’s confidence, and weaken the sense that home language practice is responsive and relationally meaningful. In this sense, ASR limitations can become communicative and affective barriers, particularly in family contexts where language transmission depends on encouragement, repetition, emotional security, and intergenerational participation. Future work should therefore distinguish more carefully between contexts in which AI acts as a direct conversational partner and those in which it functions more indirectly by structuring or supporting the surrounding communicative environment (McGonigle et al., 2024; Okur et al., 2023; Quan et al., 2026; Xu et al., 2021).

A further issue concerns governance and relational design. As AI becomes increasingly embedded in everyday communication, the question is not only how intelligent such systems are, but how they are positioned within human relational life. Recent theoretical work suggests that social risks may arise when AI is structured as an exclusive or highly personalized interlocutor, thereby displacing human-human interaction and reshaping empathy, trust, and social coordination (Ryu, 2026). Future research should therefore pay closer attention to the relational architecture of AI-mediated communication, including whether AI systems are designed to isolate users into individualized exchanges or to remain embedded within broader multi-user networks that preserve human responsibility and shared context. Examining these conditions would extend the present framework beyond interactional form to the relational sustainability of language practices in AI-mediated environments.

These directions point toward a view of language as an evolving process shaped by the interplay between human agency, communicative structure, and technological mediation. Further work along these lines would deepen the understanding of how language, affect, and identity continue to shift in increasingly complex environments.

## Data Availability

The original contributions presented in the study are included in the article/supplementary material, further inquiries can be directed to the corresponding author.
